# Microbiome and mental health in the modern environment

**DOI:** 10.1186/s40101-016-0101-y

**Published:** 2016-06-27

**Authors:** Emily Deans

**Affiliations:** Brigham and Women’s Hospital, Harvard Medical School, Boston, MA USA; Wellcare Physicians Group, 100 Morse St. Ste 105, Norwood, MA 02062 USA

**Keywords:** Microbiome, Psychobiotics, Mental health

## Abstract

A revolution in the understanding of the pathophysiology of mental illness combined with new knowledge about host/microbiome interactions and psychoneuroimmunology has opened an entirely new field of study, the “psychobiotics”. The modern microbiome is quite changed compared to our ancestral one due to diet, antibiotic exposure, and other environmental factors, and these differences may well impact our brain health. The sheer complexity and scope of how diet, probiotics, prebiotics, and intertwined environmental variables could influence mental health are profound obstacles to an organized and useful study of the microbiome and psychiatric disease. However, the potential for positive anti-inflammatory effects and symptom amelioration with perhaps few side effects makes the goal of clarifying the role of the microbiota in mental health a vital one.

## Main text

Mental health problems such as affective disorders, anxiety disorders, and psychotic disorders are not just diseases caused by psychological stressors added to genetic vulnerability, but rather full-body, inflammatory conditions related to the immune state [1–4]. In this light, the role of the microbiome, 100 trillion or so organisms that provide a barrier and a profound effect on our immune regulatory function, most notably in the large intestine [5], becomes immensely important. The state of our microbiome affects not only diseases of the gut but also autoimmune disease, obesity [6, 7], and even how well the liver metabolizes alcohol [8]. While every person’s microbiome is unique, generally healthy people share certain species, diversity, and abundance. It is clear that the microbiomes of humans living in the developed world are far different from those of hunter-gatherers living more as our ancestors did [9]. Are these observed differences in microbiota a surrogate marker for modern pressures exerted on humans? The available evidence would suggest that microbiota and mental health be viewed through the lens of physiological anthropology in the context of evolutionary medicine. Researchers have taken these findings to explore the impact of the microbiome on mental health [10].

The gut-brain connection hinges on how the microbiome influences the hypothalamic-adrenal-pituitary axis in a similar way to psychological stress, with pathogenic species of bacteria interacting with the immune and endocrine systems to create an inflammatory cascade with an increase in sympathetic reactivity [11]. These inflammatory species also have direct communication with the brain via self-produced neurotransmitters and the vagus nerve receptors in the gut, with mostly unknown significance [12, 13]. Probiotics or anti-inflammatory species, the most studied being the *Lactobacillus* and *Bifidobacteria*, can act to suppress this immune and sympathetic reaction. Demonstrations in both animal and human studies have shown that the administration of beneficial microbes can reduce both inflammation and anxiety or behavioral signs of distress [14–16]. These agents work on the same system but in a different location as chemical antidepressants, suggesting possibilities for a new “psychobiotic” class of low-side effect anti-inflammatory, anti-anxiety, and antidepressant.

The studies thus far are promising though small in scale and broad in scope. Rodent behavior [17] and levels of anxiety are influenced by probiotics, prebiotics (types of fiber that act as food for the microbiome), and fecal transplants [18]. In humans, decreased negative, ruminative thoughts have occurred in randomized controlled trials after a month on probiotics [19], while prebiotics and the administration of pseudocommensals (microbial species that do not live in the gastrointestinal tract but due to presence in water and soil have had continuously passed through the human gut in evolutionary history) have decreased anxiety [20, 21]. The rates of ADHD or autistic spectrum disorders in young teenagers supplemented with probiotics as infants dropped to 0 % compared to controls in one fascinating study [22]. In another paper, researchers were able to determine the patients with and without depression with 100 % sensitivity and 97 % specificity just by looking at the microbial genomes from fecal swabs [23]. The administration of antibiotics with antidepressants had a significant effect on psychotic depression compared to antidepressants alone [24] in a small group of inpatients. In addition, major differences have been found between the oral microbiome of people with schizophrenia vs. controls [25], and, not surprisingly, the microbiome of people with anorexia is quite different during disease behavior than in recovery [26]. Further studies postulate a role between but microbiota and Alzheimer’s dementia [27].

Having established there are some fascinating links between the microbiome and mental health, the challenges to finding safe, actionable clinical data are profound. One obvious obstacle is the sheer diversity of diagnoses of mental illness, ameliorated somewhat by the goal of addressing underlying systemic inflammation with microbiome manipulation. That goal may not be as simple as it sounds, given that the microbiome is a changeable entity reacting to local stress, food intake, sleep, and baseline conditions. One microbiome may be ideal for one person and not for another with a different diet or situation. One-size-fits-all probiotics may not show us the best clinical effect. A more bespoke approach based on microbiome sequencing and filling gaps of known guardian species such as *Bifidobacterium longum* and *Akkermnansia muciniphila* [28] may be more effective. We need to also establish the benefit/risks of low vs. high-dose probiotic supplementation and the utility of giving prebiotic fibers and probiotics together.

Food intake is a massive confounder to any study of the microbiome. Numerous studies show more traditional, whole foods are superior for mental health [29]. While optimal levels of vitamins, minerals, and certain fatty acids are key for brain functioning, these traditional diets also tend to be higher in certain fibers and prebiotics whose benefits for the microbiome could explain much of the effect of these diets on mental health [30, 31]. Clearly, we need to do more work and use the information from studies designed to more carefully discern the effects of diet, prebiotics, and probiotics on general health. Numerous one-off studies of various probiotic brands for mental disorders will leave us in a similar state to those trying to glean some wisdom from the research on omega 3 fatty acids, confused and underwhelmed.

In addition, attention must be paid to another long-term modifier of the immune system in human evolutionary history, ignored in many “psychobiotic” studies, the eukaryotic helminths [32, 33]. Humans have coevolved with helminthic infection for the entire evolutionary history of our species, and recent times are striking for their absence. Since the early 1900s such common infections such as hookworms and *Enterobius vermicularis* in the developed world have decreased tremendously [34, 35] with numerous possible downstream effects on long-term immunity and host behavior. Parasitic contribution falls alongside that of the commensal microbiome, and helminths deserve a chair at the table of microbiome research in mental health. Helminths have been used experimentally for treatment of autoimmune diseases such as Crohn’s disease and multiple sclerosis [36] with some interesting case reports of psychiatric disorders (including anxiety disorders, affective disorders, and autistic spectrum disorders) co-occuring with the autoimmune disease remitting as well [37]. It would be prudent to study helminthic therapies for neuropsychiatric conditions, particularly in those conditions such as OCD or some types of psychosis where there is a suspected autoimmune contribution [38, 39].

## Conclusions

Microbiome manipulation is an evolving tool in the armamentarium to fight mental illness. We must temper enthusiasm and marketing of any particular probiotic strain or brand with a large helping of wisdom and experience already gleaned from the many researchers becoming experts in this relatively new field of human medicine. Multidisciplinary communication among medical researchers, anthropologists, and physiologists can help us to modulate our modern environment to ameliorate the need for psychobiotics in the first place.

## References

[1] Berk M, Williams LJ, Jacka FN, O’Neil A, Pasco JA, Moylan S, Allen NB, Stuart AL, Hayley AC, Byrne ML, Maes M (2013). So depression is an inflammatory disease, but where does the inflammation come from?. BMC Med.

[2] Leonard B, Maes M (2012). Mechanistic explanations how cell-mediated immune activation, inflammation and oxidative and nitrosative stress pathways and their sequels and concomitants play a role in the pathophysiology of unipolar depression. Neurosci Biobehav Rev.

[3] Kapczinski F, Dal-Pizzol F, Teixeira AL, Magalhaies PV, Kaur-Sant’Anna M, Klamt F, Moreira JC, de Bittencourt Pasquali MA, Fries GR, Quevedo J, Gama CS, Post R (2011). Peripheral biomarkers and illness activity in bipolar disorder. J Psychiatr Res.

[4] Noto C, Ota VK, Santoro ML, Gouvea ES, Silva PN, Spindola LM, Cordeiro Q, Bressan RA, Gadelha A, Brietzke E, Belangero SI, Maes M (2015). Depression, cytokine, and cytokine by treatment interactions modulate gene expression in antipsychotic naïve first episode psychosis. Mol Neurobiol.

[5] Nagpal R, Kumar M, Yadav AK, Hemalatha R, Yadav H, Marotta F, Yamashiro Y (2015). Gut microbiota in health and disease: an overview focused on metabolic inflammation. Benef Microbes.

[6] Ridaura VK, Faith JJ, Rey FE, Cheng J, Duncan AE, Kau AL, Griffin NW, Lombard V, Henrissat B, Bain JR, Muehlbauer MJ, Ilkayeva O, Semenkovich CF, Funai K, Hayashi DK, Lyle BJ, Martini MC, Ursell LK, Clemente JC, Van Treuren W, Walters WA, Knight R, Newgard CB, Heath AC, Gordon JI (2013). Cultured gut microbiota from twins discordant for obesity modulate adiposity and metabolic phenotypes in mice. Science.

[7] Tilg H, Kaser A (2011). Gut microbiome, obesity, and metabolic dysfunction. J Clin Invest.

[8] Chen P, Schnabl B (2014). Host-microbiome interactions in alcoholic liver disease. Gut Liver.

[9] Obregon-Tito AJ, Tito RY, Metcalf J, Sankaranarayanan K, Clemente JC, Ursell LK, Zech Xu Z, Van Treuren W, Knight R, Gaffney PM, Spicer P, Lawson P, Marin-Reyes L, Trujillo-Villarroel O, Foster M, Guija-Poma E, Troncoso-Corzo L, Warinner C, Ozga AT, Lewis CM (2015). Subsistence strategies in traditional societies distinguish gut microbiomes. Nat Commun.

[10] Zhou L, Foster J (2015). Psychobiotics and the gut–brain axis: in the pursuit of happiness. Neuropsychiatr Dis Treat.

[11] Bailey MT, Dowd SE, Galley JD, Hufnagle AR, Allen RG, Lyte M (2011). Exposure to a social stressor alters the structure of the intestinal microbiota: implications for stressor-induced immunomodulation. Brain Behav Immun.

[12] Lyte M (2011). Probiotics function mechanistically as delivery vehicles for neuroactive compounds: microbial endocrinology in the design and use of probiotics. Bioessays.

[13] Forsythe P, Bienenstock J, Kunze WA (2014). Vagal pathways for microbiome-brain-gut axis communication. Adv Exp Med Biol.

[14] Tillisch K, Labus J, Kilpatrick L, Jiang Z, Stains J, Ebrat B, Guyonnet D, Legrain-Raspaud S, Trotin B, Naliboff B, Mayer EA (2013). Consumption of fermented milk product with probiotic modulates brain activity. Gastroenterology.

[15] Cryan JF, Dinan TG (2012). Mind-altering microorganisms: the impact of the gut microbiota on brain and behaviour. Nat Rev Neurosci.

[16] Messaoudi M, Lalonde R, Violle N, Javelot H, Desor D, Nejdi A, Bisson JF, Rougeot C, Pichelin M, Cazaubiel M, Cazaubiel JM (2011). Assessment of psychotropic-like properties of a probiotic formulation (Lactobacillus helveticus R0052 and Bifidobacterium longum R0175) in rats and human subjects. Br J Nutr.

[17] Bercik P, Denou E, Collins J, Jackson W, Lu J, Jury J, Deng Y, Blennerhassett P, Macri J, McCoy KD, Verdu EF, Collins SM (2011). The intestinal microbiota affect central levels of brain-derived neurotropic factor and behavior in mice. Gastroenterology.

[18] Bravo JA, Forsythe P, Chew MV, Escaravage E, Savignac HM, Dinan TG, Bienenstock J, Cryan JF (2011). Ingestion of Lactobacillus strain regulates emotional behavior and central GABA receptor expression in a mouse via the vagus nerve. Proc Natl Acad Sci U S A.

[19] Steenbergen L, Sellaro R, van Hemert S, Bosch JA, Colzato LS (2015). A randomized controlled trial to test the effect of multispecies probiotics on cognitive reactivity to sad mood. Brain Behav Immun.

[20] Silk DBA, Davis A, Vulevic J, Tzortzis G, Gibson GR (2009). Clinical trial: the effects of a trans-galactooligosaccharide prebiotic on faecal microbiota and symptoms in irritable bowel syndrome. Aliment Pharmacol Ther.

[21] O’Brien ME, Anderson H, Kaukel E, O’Byrne K, Pawlicki M, Von Pawel J, Reck M (2004). SRL172 (killed Mycobacterium vaccae) in addition to standard chemotherapy improves quality of life without affecting survival in patients with advanced non-small-cell lung cancer: phase III results. Ann Oncol.

[22] Pärtty A, Kalliomäki M, Wacklin P, Salminen S, Isolauri E (2015). A possible link between early probiotic intervention and the risk of neuropsychiatric disorders later in childhood: a randomized trial. Pediatr Res.

[23] Naseribafrouei A, Hestad K, Avershina E, Sekelja M, Linløkken A, Wilson R, Rudi K (2014). Correlation between the human fecal microbiota and depression. Neurogastroenterol Motil.

[24] Miyaoka T, Wake R, Furuya M, Liaury K, Ieda M, Kawakami K, Tsuchie K, Taki M, Ishihara K, Araki T, Horiguchi J (2012). Minocycline as adjunctive therapy for patients with unipolar psychotic depression: an open-label study. Prog Neuropsychopharmacol Biol Psychiatry.

[25] Castro-Nallar E, Bendall ML, Pérez-Losada M, Sabuncyan S, Severance EG, Dickerson FB, Schroeder JR, Yolken RH, Crandall KA (2015). Composition, taxonomy and functional diversity of the oropharynx microbiome in individuals with schizophrenia and controls. PeerJ.

[26] Kleiman SC, Watson HJ, Bulik-Sullivan EC, Huh EY, Tarantino LM, Bulik CM, Carroll IM (2015). The intestinal microbiota in acute anorexia nervosa and during renourishment: relationship to depression, anxiety, and eating disorder psychopathology. Psychosom Med.

[27] Bester J, Soma P, Kell DB, Pretorius E (2015). Viscoelastic and ultrastructural characteristics of whole blood and plasma in Alzheimer-type dementia, and the possible role of bacterial lipopolysaccharides (LPS). Oncotarget.

[28] Dao MC, Everard A, Aron-Wisnewsky J, Sokolovska N, Prifti E, Verger EO, Kayser BD, Levenez F, Chilloux J, Hoyles L, Dumas ME, Rizkalla SW, Doré J, Cani PD, Clément K, MICRO-Obes Consortium (2015). Akkermansia muciniphila and improved metabolic health during a dietary intervention in obesity: relationship with gut microbiome richness and ecology. Gut.

[29] Rahe C, Unrath M, Berger K (2014). Dietary patterns and the risk of depression in adults: a systematic review of observational studies. Eur J Nutr.

[30] Dash S, Clarke G, Berk M, Jacka FN (2015). The gut microbiome and diet in psychiatry: focus on depression. Curr Opin Psychiatry.

[31] O’Keefe SJ, Li JV, Lahti L, Ou J, Carbonero F, Mohammed K, Posma JM, Kinross J, Wahl E, Ruder E, Vipperla K, Naidoo V, Mtshali L, Tims S, Puylaert PG, DeLany J, Krasinskas A, Benefiel AC, Kaseb HO, Newton K, Nicholson JK, de Vos WM, Gaskins HR, Zoetendal EG (2015). Fat, fibre and cancer risk in African Americans and rural Africans. Nat Commun.

[32] Rook GA, Raison CL, Lowry CA (2014). Microbiota, immunoregulatory old friends and psychiatric disorders. Adv Exp Med Biol.

[33] Raison CL, Lowry CA, Rook GA (2010). Inflammation, sanitation, and consternation: loss of contact with coevolved, tolerogenic microorganisms and the pathophysiology and treatment of major depression. Arch Gen Psychiatry.

[34] Elliott DE, Weinstock JV (2012). Helminth–host immunological interactions: prevention and control of immune-mediated diseases. Ann N Y Acad Sci.

[35] Chang TK, Liao CW, Huang YC, Chang CC, Chou CM, Tsay HC, Huang A, Guu SF, Kao TC, Fan CK (2009). Prevalence of Enterobius vermicularis infection among preschool children in kindergartens of Taipei City, Taiwan in 2008. Korean J Parasitol.

[36] Fleming JO, Isaak A, Lee JE, Luzzio CC, Carrithers MD, Cook TD, Field AS, Boland J, Fabry Z (2011). Probiotic helminth administration in relapsing–remitting multiple sclerosis: a phase 1 study. Mult Scler.

[37] Cheng AM, Jaint D, Thomas S, Wilson JK, Parker W (2015). Overcoming evolutionary mismatch by self-treatment with helminths: current practices and experience. J Evol Med.

[38] Nicolini H, López Y, Genis-Mendoza AD, Manrique V, Lopez-Canovas L, Niubo E, Hernández L, Bobes MA, Riverón AM, López-Casamichana M, Flores J, Lanzagorta N, De la Fuente-Sandoval C, Santana D (2015). Detection of anti-streptococcal, antienolase, and anti-neural antibodies in subjects with early-onset psychiatric disorders. Actas Esp Psiquiatr.

[39] Steiner J, Walter M, Glanz W, Sarnyai Z, Bernstein HG, Vielhaber S, Kästner A, Skalej M, Jordan W, Schiltz K, Klingbeil C, Wandinger KP, Bogerts B, Stoecker W (2013). Increased prevalence of diverse N-methyl-D-aspartate glutamate receptor antibodies in patients with an initial diagnosis of schizophrenia: specific relevance of IgG NR1a antibodies for distinction from N-methyl-D-aspartate glutamate receptor encephalitis. JAMA Psychiatry.

